# Drug-Induced Acute Pancreatitis After Long-Term Sulfasalazine Therapy

**DOI:** 10.7759/cureus.10441

**Published:** 2020-09-14

**Authors:** Shehriyar Mehershahi, Asim Haider, Danial Shaikh, Hafsa Abbas, Ariyo Ihimoyan

**Affiliations:** 1 Gastroenterology, BronxCare Health System, Bronx, USA; 2 Internal Medicine, BronxCare Health System, Bronx, USA; 3 Medicine/Gastroenterology, BronxCare Health System, Bronx, USA

**Keywords:** sulfasalazine, pancreatitis, alcohol, gallstones, drug

## Abstract

The list of drugs associated with acute pancreatitis is increasing with each passing year. Nevertheless, knowledge of drug-induced pancreatitis (DIP) are often curtailed by the limited availability of evidence needed to implicate given agents, especially for non-prescription medications. Indeed, the majority of available data are derived from case reports, case series, or case-control studies. We present a case chemically and radiologically proven pancreatitis in a 43-year-old female who was on sulfasalazine as a maintenance therapy for ulcerative colitis.

## Introduction

Acute pancreatitis (AP) is the leading gastrointestinal cause of hospitalization in the United States [1]. The reported annual incidence of AP in the United States ranges from 4.9 to 35 per 100,000 population. The incidence of AP is increasing worldwide due to increased rates of obesity and gallstones [2]. Pancreatitis due to medications is rare (<5%). In 1955, Zion et al, were the first to report a case of drug-induced acute pancreatitis (DIP) by describing a hemorrhagic pancreatitis associated with cortisone therapy [3]. Since then, there have been numerous reports of AP occurring in association with drugs.

It has become increasingly recognized that DIP represents an essential and growing though often inconspicuous cause of AP. DIP is a diagnosis of exclusion. Ruling out other causes of AP can be challenging, especially in patients with multiple underlying comorbidities and utilization of numerous prescriptions including herbal medications. Prevention of DIP requires up-to-date knowledge of drugs with the most reliable evidence connecting their use to the development of pancreatitis. While interstitial and necrotizing pancreatitis have a mortality rate of 5% and 17%, respectively, the prognosis of DIP is generally excellent, and mortality is low [4].

## Case presentation

A 43-year-old female presented to the emergency department with acute onset of abdominal pain for the last 12 hours. The pain was located in the epigastric region and the left upper quadrant of the abdomen, 8/10 in intensity, sharp in nature, radiating to back associated with two episodes of non-bilious and non-bloody vomiting, no aggravating or relieving factors. The patient medical history was significant for ulcerative colitis. Her past surgical history was significant for three cesarean sections. She reported that she used to drink alcoholic beverages occasionally (one to two times per month) but stopped drinking five years ago. She denied any history of smoking or any other toxic habits. Her only medication was oral sulfasalazine two grams per day past 18 months. She denied any known allergies. The patient denied using herbal or weight losing medication

**Figure 1:**
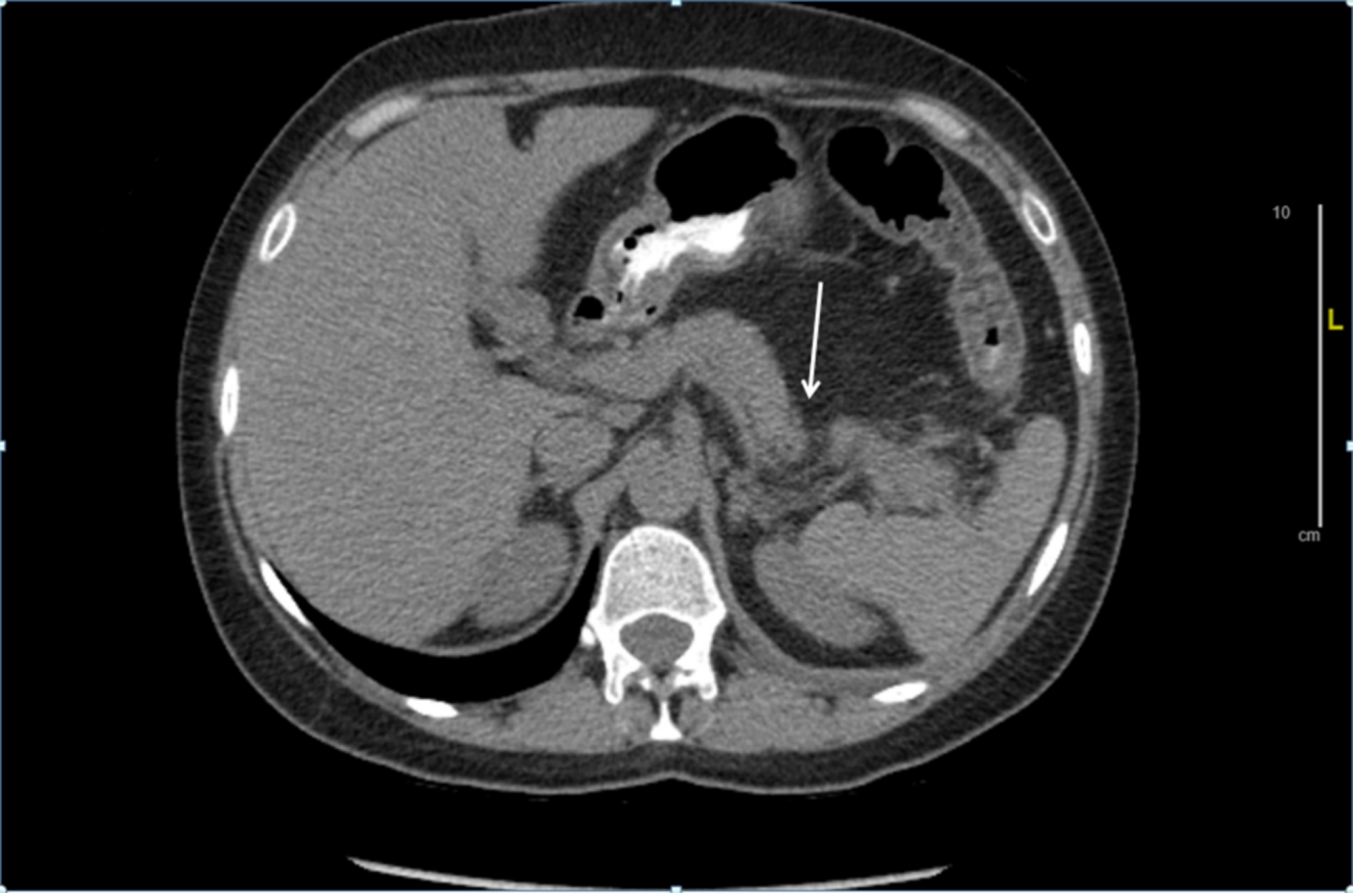
CT scan of the abdomen showing infiltration of mesenteric fat around the tail of the pancreas suggestive of acute pancreatitis

Her initial vitals were a pulse rate of 94 bpm, blood pressure of 126/86 mmHg, respiratory rate of 17 breaths per minute, and temperature of 98.6˚F. On physical examination, the abdomen was soft with tenderness in the epigastric region, and bowel sounds were normal. Her initial labs showed an alanine aminotransferase (ALT) of 62 units/L (5-40 units/L), aspartate transaminase (AST) 164 units/L (9-36 units/L), alkaline phosphatase 98 units/L (42-98 units/L), total bilirubin 1.5 mg/dL (0.2-1.2 mg/dL), conjugated bilirubin 0.5 mg/dL (0.0-0.3 mg/dL), serum lipase 419 U/L (<60 U/L), serum ethanol level <10 mg/dL (<10 mg/dL), serum triglyceride level 88 mg/dL (45-150 mg/dL), serum calcium 8.7 mg/dL (8.5-10.5 mg/dL), albumin 3.8 g/dL (3.2-4.8 mg/dL), blood urea nitrogen (BUN) 11 mg/dL (6-20 mg/dL), and serum creatinine 0.5 mg/dL (0.5-1.5 mg/dL). Ultrasound of the abdomen showed a normal size and texture of liver, normal gallbladder with no gallstones, and bile duct was 3 mm in size. A CT of the abdomen with oral contrast showed findings suggestive of AP without any pancreatic necrosis or pseudocyst formation, no pancreatic duct, or common bile duct dilatation (Figure 1).

Initially, during admission, sulfasalazine was continued as maintenance therapy for ulcerative colitis with no improvement in symptoms. The patient continued to complain of abdominal pain and nausea. Later on, sulfasalazine was discontinued and the patient started feeling better. Two days after discontinuation of sulfasalazine, she was able to tolerate a clear liquid diet, and shortly after her diet was advanced to regular. Symptoms had resolved by day 5, and the patient was discharged safely.

## Discussion

AP is caused by a wide variety of etiologies. Gallstones are the most common cause of AP accounting for 40% to 70% of cases. However, only 3% to 7% of patients with gallstones develop pancreatitis [5]. Alcohol is responsible for approximately 25% to 35% of cases of AP in the United States . It is estimated that heavy drinking of ethanol (>150 g/day or about 10-11 standard U.S. drinks) for a minimum of 6-12 years is generally required to produce symptomatic pancreatitis. The risk of developing the disease is proportional to the duration and amount of alcohol consumption [6]. Hypertriglyceridemia (usually levels above 1,000 mg/dL) may account for 1% to 14% of cases of AP [7]. 

Epidemiologic data suggest the risk of pancreatitis is highest for mesalazine (hazard ratio [HR] 3.5), azathioprine (HR 2.5), and simvastatin (HR 1.8) [8]. DIP is classified (class Ia, Ib, II, III, IV) based on the number of cases reported, demonstration of a consistent latency period (time from initiation of the drug to development of pancreatitis), and reaction with rechallenge [9].

Class 1 drugs were subdivided into Ia and Ib. Class Ia includes at least one documented case following re-exposure and excluding all other causes, such as alcohol, gallstone, hypertriglyceridemia, and other drugs. Class Ib drugs are alike class Ia. However, in this class, potential causes of AP were not ruled out or clearly present. Class II drugs include at least four cases reported in literature to be in this group, and in which there is a consistent latency in at least 75% or more of the cases that have been reported. Class I and II drugs have the highest potential for causing AP. Class III drugs are weaker then previous two classes, and do not have a consistent latency period or rechallenge data. Finally, class IV drugs include drugs not fitting into rest of the mentioned classes, and have a single case report published in medical literature, without rechallenge data. Latencies were classified to short (latency of <24 hours), intermediate (latency of 1-30 days), and long (latency >30 days) [9,10]. The symptoms must resolve after the drug is discontinued to classify the episode of AP as drug-induced [9].

If DIP is suspected, the implicated drug should be discontinued. The resolution of pancreatitis after discontinuation of the drug increases the suspicion of DIP. However, this connection can be challenging to establish as the resolution of pancreatitis may be linked coincidentally with the cessation of the implicated drug. The only way to establish a definite diagnosis is to rechallenge with the offending drug resulting in the recurrence of pancreatitis, although this may not be feasible all the times [9,10].

Various theories have been proposed to understand the mechanism of DIP. These include immunological reactions (aminosalicylates, sulfonamides), ischemia (diuretics, azathioprine), accumulation of a toxic metabolite (e.g., valproic acid, didanosine, pentamidine, tetracycline), direct toxic effect (e.g., diuretics, sulfonamides), intravascular thrombosis (e.g., estrogen), and an increased viscosity of pancreatic juice (e.g., diuretics and steroids) [11,12]. 

Salicylazosulfapyridine (also known as sulfasalazine, SSZ) is a prodrug composed of 5-aminosalicylic acid (5-ASA) linked to sulfapyridine through an azo bond [13]. Sulfapyridine is a sulfonamide antibacterial medication. There have been some case reports about the association of sulfonamides with AP. Among sulfonamides, sulfamethoxazole and sulfapyridine have been most associated with pancreatitis [14,15]. Pure 5-ASA derivative mesalamine (a 5-ASA moiety alone without any sulfa molecule) has also been associated with pancreatitis [16]. Interestingly, both oral and enema 5-ASA preparations have been implicated in causing pancreatitis [17]. The pathogenic mechanism of sulfasalazine-induced pancreatitis remains unknown. The proposed pathogenic mechanism is increased permeability of the pancreatic duct due to the direct effect of salicylic acid [18]. Also, an allergic hypersensitivity mechanism has been proposed in some cases [16].

Finally, there are reported cases of AP after short term and after long term exposure to 5-ASA derivatives. One study reported two cases of proven AP occurring 2 and 14 days, respectively, after oral 5-aminosalicylic acid therapy was instituted for inflammatory bowel disease [18]. Other studies have reported that AP can also occur after long-term exposure to 5-ASA derivatives in some cases [19].

## Conclusions

Both molecules of sulfasalazine (5-aminosalicylic acid and sulfapyridine) should be considered class I drugs associated with pancreatitis. The probable mechanism includes immunological reaction vs direct toxic effect. The onset of pancreatitis can occur a few days after exposure or may happen after many years of exposure. There are not many cases reported in literature with long-term use of sulfasalazine causing AP. This case helps in increasing awareness and minimize excessive unnecessary investigations, patient anxiety, and health care costs.

## References

[1] Peery AF, Dellon ES, Lund J (2012). Burden of gastrointestinal disease in the United States: 2012 update. Gastroenterology.

[2] Toouli J, Brooke-Smith M, Bassi C (2002). Guidelines for the management of acute pancreatitis. J Gastroenterol Hepatol.

[3] Zion MM, Goldberg B, Suzman MM (1955). Corticotrophin and cortisone in the treatment of scleroderma. Q J Med.

[4] Lankisch PG, Dröge M, Gottesleben F (1995). Drug induced acute pancreatitis: incidence and severity. Gut.

[5] Moreau JA, Zinsmeister AR, Melton LJ 3rd, DiMagno EP (1988). Gallstone pancreatitis and the effect of cholecystectomy: a population-based cohort study. Mayo Clin Proc.

[6] Yang AL, Vadhavkar S, Singh G, Omary MB (2008). Epidemiology of alcohol-related liver and pancreatic disease in the United States. Arch Intern Med.

[7] Wan J, He W, Zhu Y (2017). Stratified analysis and clinical significance of elevated serum triglyceride levels in early acute pancreatitis: a retrospective study. Lipids Health Dis.

[8] Nitsche CJ, Jamieson N, Lerch MM, Mayerle JV (2010). Drug induced pancreatitis. Best Pract Res Clin Gastroenterol.

[9] Badalov N, Baradarian R, Iswara K, Li J, Steinberg W, Tenner S (2007). Drug-induced acute pancreatitis: an evidence-based review. Clin Gastroenterol Hepatol.

[10] Jones MR, Hall OM, Kaye AM, Kaye AD (2015). Drug-induced acute pancreatitis: a review. Ochsner J.

[11] Sadr-Azodi O, Mattsson F, Bexlius TS, Lindblad M, Lagergren J, Ljung R (2013). Association of oral glucocorticoid use with an increased risk of acute pancreatitis: a population-based nested case-control study. JAMA Intern Med.

[12] Weissman S, Aziz M, Perumpail RB, Mehta TI, Patel R, Tabibian JH (2020). Ever-increasing diversity of drug-induced pancreatitis. World J Gastroenterol.

[13] Smedegård G, Björk J (1995). Sulphasalazine: mechanism of action in rheumatoid arthritis. Br J Rheumatol.

[14] Brazer SR, Medoff JR (1988). Sulfonamide-induced pancreatitis. Pancreas.

[15] Bartels RH, van der Spek JA, Oosten HR (1992). Acute pancreatitis due to sulfamethoxazole-trimethoprim. South Med J.

[16] Fernandez J, Sala M, Panes J, Feu F, Navarro S, Teres J (1997). Acute pancreatitis after long term 5-aminosalicylic acid therapy. Am J Gastroenterol.

[17] Fiorentini MT, Fracchia M, Galatola G, Barlotta A, de la Pierre M (1990). Acute pancreatitis during oral 5-aminosalicylic acid therapy. Dig Dis Sci.

[18] Isaacs KL, Murphy D (1990). Pancreatitis after rectal administration of 5-aminosalicylic acid. J Clin Gastroenterol.

[19] Ouakaa-Kchaou A, Gargouri D, Kochlef A, Bibani N, Elloumi H, Trad D, Kharrat J (2014). Acute pancreatitis secondary to long-term 5-aminosalicylic acid therapy in a patient with ulcerative colitis: a case report. Tunis Med.

